# AI-Reinforced Wearable Sensors and Intelligent Point-of-Care Tests

**DOI:** 10.3390/jpm14111088

**Published:** 2024-11-01

**Authors:** Ghita Yammouri, Abdellatif Ait Lahcen

**Affiliations:** 1 Chemical Analysis & Biosensors, Process Engineering and Environment Laboratory, Faculty of Science and Techniques, Hassan II University of Casablanca, Mohammedia 28806, Morocco; ghitayammouri@gmail.com; 2 Center for Bioelectronics, Old Dominion University, Norfolk, VA 23508, USA

**Keywords:** artificial intelligence, wearable sensors, point-of-care testing, deep learning, personalized medicine, biosensors

## Abstract

Artificial intelligence (AI) techniques offer great potential to advance point-of-care testing (POCT) and wearable sensors for personalized medicine applications. This review explores the recent advances and the transformative potential of the use of AI in improving wearables and POCT. The integration of AI significantly contributes to empowering these tools and enables continuous monitoring, real-time analysis, and rapid diagnostics, thus enhancing patient outcomes and healthcare efficiency. Wearable sensors powered by AI models offer tremendous opportunities for precise and non-invasive tracking of physiological conditions that are essential for early disease detection and personalized treatments. AI-empowered POCT facilitates rapid, accurate diagnostics, making these medical testing kits accessible and available even in resource-limited settings. This review discusses the key advances in AI applications for data processing, sensor fusion, and multivariate analytics, highlighting case examples that exhibit their impact in different medical scenarios. In addition, the challenges associated with data privacy, regulatory approvals, and technology integrations into the existing healthcare system have been overviewed. The outlook emphasizes the urgent need for continued innovation in AI-driven health technologies to overcome these challenges and to fully achieve the potential of these techniques to revolutionize personalized medicine.

## 1. Introduction

The timely and accurate diagnosis of health conditions is of high importance within any healthcare system for effective disease management. This helps to accurately monitor disease progression and alleviates the financial, psychological, and social stress experienced by patients [1,2]. In this context, early detection of health conditions is crucial as it greatly affects the selected treatment plan and, as a result, improves health outcomes. Personalized and precision medicine relies mainly on the continuous monitoring of health conditions by obtaining a rapid and fast diagnosis using point-of-care (PoC) [3]. Wearable biosensor devices have recently evolved as emerging analytical-tool technologies used for rapid in vitro diagnostics testing and have the potential to ensure more timely and customized medical care [4,5]. Despite the great progress that has been made so far in these biosensor technologies, there is still a need to boost the analytical capabilities of these devices [6]. To do so, artificial intelligence (AI) techniques offer a plethora of opportunities and a promise toward the reinforcement of wearable sensing and PoC testing to meet the increasing demands for personalized and precision medicine [7,8].

Recently, huge interest has been exponentially growing in favor of integrating AI techniques, such as machine learning (ML) and deep neural networks with wearable devices, as well as PoC-based diagnostic tools [9,10]. This allows for many advantages, such as the capturing of multivariate data with the on-body sensors and POCT devices. Beyond simply aggregating data, AI techniques can help to reduce the number of experiments needed to prepare the sensor and help to predict the best conditions for sensor preparation for a certain disease [11,12]. Such smart and intelligent sensing devices can track health status in real time while minimizing discomfort, allowing for early intervention in case of a medical emergency. This can also facilitate remote monitoring and telemedicine, which is becoming increasingly needed and relevant, especially in recent years [13].

AI innovations can serve to improve diagnostic testing rapidly at the level of PoC by making sample analysis more automated, thus providing faster and more accurate analysis outside of traditional laboratories [14]. A great and successful example of this is the intelligent readers and assays powered by AI that can be used to detect cell and biomarker morphologies in minutes [15]. These technologies are making sophisticated testing accessible in low-resource settings and enable molecular diagnostics at the site of care. Efforts to combine AI with wearable sensors and PoC diagnostics greatly correlate with the objectives of personalized medicine. Smart biosensors allow for continuous physiological monitoring to detect medical needs as they emerge based on an individual’s unique biology. Meanwhile, rapid diagnostic insights at the PoC facilitate the timely application of precision therapies. However, while promising, AI-reinforced wearable and PoC technologies remain in their infancy and early development. The key challenges could include different aspects, such as the validation of AI-based models, the integration of the AI techniques into reusable medical devices, and regulatory approval by government agencies, as well as the adoption of these technologies by the medical community [16].

There have been many review papers that cover the topic of AI and smart wearable sensors in recent years [17,18,19]. This shows that this field is trending and is one of the hot topics in the research field. However, there is still a need for a comprehensive review manuscript that incorporates smart wearable biosensors and PoC devices. While the recent literature has extensively covered various aspects of AI in healthcare, wearable sensors, and POCT (Table 1), our proposed review uniquely focuses on the intersection and synergy of these technologies. Unlike previous reviews that often address these topics separately, we aim to provide a comprehensive analysis of AI-reinforced wearable sensors and intelligent POCT. This integrated approach not only bridges the gap in the current literature but also offers a more forward-looking perspective on how AI enhances both wearable sensors and PoC diagnostics.

In this review paper, we discussed the use of AI-enabled biosensors and intelligent rapid diagnostic devices for personalized medicine applications. Technical approaches, advantages, and limitations of this field have been discussed and analyzed. In the end, we identified future opportunities and current challenges for further advancing AI-reinforced wearable sensors and PoC diagnostics.

## 2. Wearable Sensors for Personalized Health Monitoring

Wearable sensors and biosensors have become powerful tools used to continuously monitor physiological signals, thus empowering the personalized medicine field. Processing smart data that is acquired by these sensors using AI technology can provide a clear and deep understanding of an individual’s health and serve to make suitable clinical decisions. This section of the manuscript overviews the key technologies in AI methods used in wearable sensors for personalized health monitoring.

### 2.1. Biosensor Technologies for Continuous Physiological Monitoring

Biosensors are analytical devices that are crucial for continuous physiological monitoring, integrating a bioreceptor for target analyte recognition, a transducer for signal conversion, electronics for processing, and a display unit for data presentation [28,29]. This field has seen significant interest due to wearable technology advancements that enable non-invasive biomarker monitoring in real time [30].

**Figure 1:**
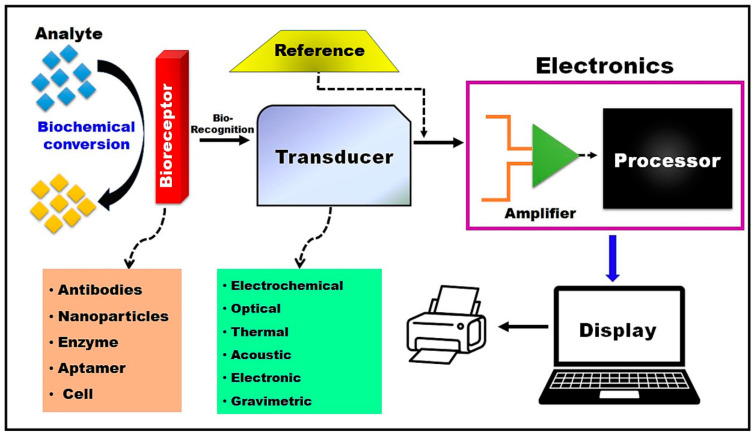
General principle of biosensors. Reused with permission from Elsevier publisher [34].

Wearable biosensors, particularly sweat and tear-based ones, offer personalized POCT by instantly quantifying the biomarkers in bodily fluids [20,31]. Body-based biomolecular sensors, including wearables, implants, and consumables, allow for comprehensive health monitoring, with glucose sensors leading the way and advancements enabling the sensing of various analytes [32]. Integrating biosensors with wireless systems and big data analytics has enhanced personalized healthcare, offering efficient and tailored monitoring of physiological parameters for improved well-being [33]. Biosensors, those remarkable devices that bridge the gap between biology and technology, are present in many different forms (Figure 1). Electrochemical biosensors measure electrical changes resulting from biochemical reactions, such as glucose monitoring. Optical biosensors rely on light-based detection methods, while piezoelectric biosensors use materials that generate an electrical charge when subjected to mechanical stress. Finally, thermal biosensors detect temperature changes due to binding events.

These biosensors play an essential role in fields ranging from medical diagnostics to environmental monitoring, making them indispensable tools for scientific and clinical research [35]. They are transforming the diagnosis of disease in healthcare by offering rapid and accurate detection of specific biomarkers with high sensitivity and specificity, thus improving the accuracy, sustainability, and efficiency of diagnostic tools [36]. These devices, which convert molecular recognition of target analytes into measurable signals, have evolved to be compact, user-friendly, and cost-effective for PoC testing, enhancing the speed and reliability of disease diagnosis [37,38]. They utilize various technologies, such as enzyme-based sensors, immunoassays, photo-biosensors, tissue-based sensors, DNA biosensors, and nano-biosensors, to detect biological markers and provide real-time insights into health conditions [39,40]. Furthermore, wearable biosensors play a crucial role in continuous health monitoring, enabling personalized medicine, early disease detection, and improved health outcomes by integrating advanced technologies like AI, ML, and microfluidics [41,42]. These advancements in biosensor technology, coupled with the development of novel molecular markers, offer a promising avenue for enhancing disease diagnosis and patient care in healthcare [43].

The development of biosensors in healthcare faces several challenges and future directions. Miniaturization and power consumption are crucial for implantable biosensors to reduce discomfort and ensure reliable functionality. Biocompatibility and long-term stability are essential for biosensors in the in vivo environment to minimize immune responses and ensure their sustained performance [44]. Data accuracy and reliability are critical, particularly in whole blood analysis, where electrochemical sensors offer rapid, sensitive, and specific detection capabilities [45]. Integration with technologies like the Internet of Things (IoT) and AI presents a promising avenue, with advancements in wearable biosensing technologies utilizing AI for precise disease diagnosis and personalized medicine [46]. Overcoming these challenges and focusing on improving sensitivity, selectivity, reproducibility, and stability will drive the future development of biosensors, opening new possibilities for biomedical applications.

### 2.2. AI for Sensor and Biosensor Data Processing and Health Analysis

The integration of AI in processing sensors and biosensor data is increasingly vital for health analysis due to the growing volume and complexity of data generated by modern wearable devices and medical technologies. Recent advancements in AI have enabled the effective analysis of vast datasets from various sources, including wearable sensors and medical imaging, which are essential for personalized medicine and PoC diagnostics [47,48]. AI techniques are pivotal in processing the data from sensors and biosensors in various healthcare applications. ML and deep learning (DL) are extensively used for analyzing medical images and wearable sensor data, thereby enhancing disease diagnosis and prediction accuracy. Deep learning, as a subset of ML, leverages complex neural networks to analyze data and solve intricate problems in healthcare. Key techniques include Convolutional Networks (CNNs) for medical image analysis, Recurrent Neural Networks (RNNs) for processing sequential health data, and Long Short-Term Memory networks (LSTMs) for predicting patient outcomes [49,50,51].

ML techniques, such as neural networks and Support Vector Machines (SVMs), automate forecasting and diagnosis processes, while DL, particularly CNNs, excels in image analysis without requiring expert feature extraction [52]. Natural Language Processing (NLP) is increasingly being integrated into electronic medical records, improving clinical data analysis and supporting clinical decision-making [24]. Additionally, Computer Vision techniques are crucial in processing images from medical imaging devices, significantly reducing diagnostic errors and improving efficiency [52]. Finally, RNNs and Long Short-Term Memory (LSTM) networks are particularly effective for analyzing time-series data generated by wearable sensors, which enable the monitoring of physiological signals and disease progression over time [46]. These models excel in capturing temporal dependencies, which is essential for applications like continuous glucose monitoring and psychological disorder detection [53,54].

**Figure 2:**
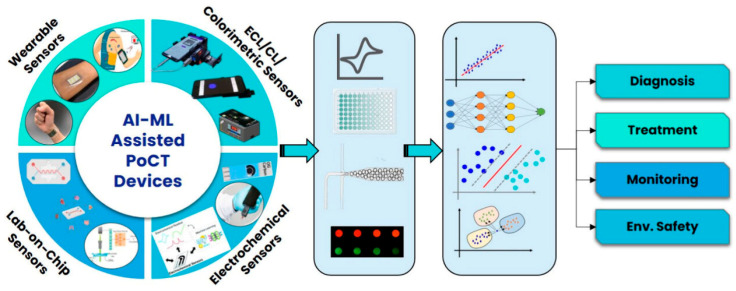
A schematic illustration of the AI/ML-assisted POCT-based biosensing devices used for clinical decision-making. Reused with permission from ACS publisher [2].

As shown in Figure 2, AI/ML has been widely used recently in POCT devices as it can help to make more accurate clinical decisions [2]. The flow from various sensor inputs, including electrochemical, wearables, colorimetric, and lab-on-chip, through data processing and ML, could lead to diagnosis, treatment, and safety. This approach demonstrates how AI and ML can improve on-site medical testing.

The integration of these AI techniques in sensor data processing is transforming healthcare delivery, enabling personalized medicine and timely interventions [11,46]. Furthermore, the shift towards real-time data collection and analysis through portable devices allows for immediate health monitoring and intervention, bypassing traditional laboratory methods [55]. As healthcare data continues to expand, the need for sophisticated AI algorithms becomes critical to manage and derive actionable insights from this complex data, which will ultimately revolutionize diagnostic practices and treatment strategies [55,56].

As an example of the application of AI and biosensing technology in biomedical cases, we identify novel approaches that combine electrochemical biosensors with AI to enhance the detection of dopamine in complex biological samples, such as cerebrospinal fluid (CSF). Traditional methods struggle with selectivity due to interference from other electroactive species, like ascorbic acid (AA) and uric acid (UA), which can significantly affect dopamine quantification. A study employed an embedded AI model, specifically TinyML, to analyze square-wave voltammetry (SWV) data, allowing for the differentiation between dopamine and its interfering compounds without the need for time-consuming surface modifications. The results indicated that the AI-enhanced biosensor achieved an impressive accuracy of 98.1% in distinguishing between the contaminated and uncontaminated dopamine samples, demonstrating the potential for real-time monitoring in clinical settings. This integration not only improves the reliability of neurotransmitter detection but also paves the way for the development of smart, portable diagnostic tools that can adapt and learn from new data over time, ultimately contributing to better screening for neurodegenerative diseases [57]. Another study presented a novel approach to prostate cancer (PCa) screening using a urinary multimarker biosensor combined with AI analysis. This method leverages the passive diffusion of biomarkers from prostate cancer cells into urine, allowing for noninvasive testing. The biosensor employs four pathophysiologically uncorrelated biomarkers, PSMA, ENG, ERG, and ANXA3, measured through a dual-gate, field-effect transistor biosensor with antibody conjugation for each biomarker. The results demonstrated that single-biomarker analysis yielded an average accuracy of only 62.9%, missing nearly half of the PCa cases. However, when ML algorithms, specifically Random Forest (RF) and Neural Networks (NNs), were applied to the multimarker data, the accuracy significantly improved, achieving over 99% accuracy with the optimal combination of biomarkers. The study highlighted that increasing the number of biomarkers generally enhanced screening performance, although the inclusion of certain biomarkers, like ANXA3, could decrease accuracy. Ultimately, the research indicates that this AI-assisted multimarker sensing platform has the potential to revolutionize PCa screening by providing rapid, accurate results using just a drop of urine, thus addressing the limitations of traditional serum PSA tests and reducing unnecessary biopsies [58].

A study presented a novel fluorometric aptasensor combined with IA designed for the simultaneous detection of lysozyme (LYS), which is considered a key biomarker for identifying several diseases, such as sarcoidosis, monocytic or myelomonocytic leukemia, and bronchopulmonary dysplasia, and adenosine triphosphate (ATP), and is considered an indicator of cell viability and many diseases, such as Parkinson’s disease, malignant tumors, and Alzheimer’s disease. This fluorometric aptasensor showcases the integration of biosensors and AI in health applications. This dual-functional aptasensor utilizes cobalt oxyhydroxide (CoOOH) nanosheets as a fluorescence quencher and carbon dots (CDs) as fluorophores, enabling the effective monitoring of these biomarkers, which is crucial for disease detection, particularly in cancer research. The method leverages fluorescence resonance energy transfer, allowing for high selectivity and sensitivity in detecting small molecules and proteins. However, the overlapping fluorescence spectra of the two types of CDs posed a challenge for simultaneous analysis. To address this, the study employed least squared support vector machine (LS-SVM) techniques, a form of AI, to deconvolute the overlapping spectra, enhancing the accuracy of the detection process. The results demonstrated that, under optimal conditions, the detection limits for ATP and LYS were 4.0 and 1.8 nmol L, respectively, indicating the method’s effectiveness in biological sample monitoring. This innovative approach not only highlights the potential of biosensors in health diagnostics but also emphasizes the role of AI in resolving complex analytical challenges, paving the way for advanced disease detection methodologies [59].

In a separate study, a novel noninvasive sensor for detecting glucose and fructose levels was developed using surface-enhanced infrared absorption (SEIRA) spectroscopy combined with principal component analysis (PCA) as an ML algorithm for data evaluation. The sensor employs linear gold nanoantennas fabricated on IR-transparent substrates that are designed to resonate at the molecular vibrations of glucose and fructose. This allows for the reliable detection of concentrations as low as 10 g/L (55 mM), which is relevant for monitoring blood glucose levels in patients with diabetes. The results demonstrated that the sensor can effectively distinguish between glucose and fructose in mixed solutions, overcoming challenges related to crosstalk from other chemical species. The integration of PCA enhances the analysis by autonomously identifying patterns in the vibrational data, making it suitable for real-time monitoring of physiological glucose levels in bodily fluids, such as interstitial fluid or teardrops. This innovative approach not only improves the sensitivity and specificity of glucose detection but also paves the way for future advancements in noninvasive biosensing technologies, potentially transforming diabetes management and other health monitoring applications [60]. In other studies, leveraged sensors and AI were employed to advance biomedical diagnostics for early-stage lung cancer. Surface-enhanced Raman spectroscopy (SERS) sensors were used to capture signals from exosomes in the blood, which are small vesicles associated with cancer biomarkers. Deep learning was used to analyze these SERS signals and to train a model to distinguish between normal and lung cancer cell exosomes with 95% accuracy. When tested on 43 patients, the model identified a high similarity between plasma exosomes from 90.7% of cancer patients and lung cancer cell exosomes, with the similarity correlating with cancer progression. The combination of SERS sensors and AI offers a promising method for noninvasive early-stage lung cancer diagnosis, as evidenced by the model’s high accuracy and area under the curve (AUC) scores of 0.912 for the entire cohort and 0.910 for stage I patients [61].

AI-based techniques are revolutionizing mental health support and psychological well-being in several key ways. Virtual counseling, powered by ML and NLP, is making mental health support more accessible and affordable, reaching individuals who might otherwise avoid traditional therapy due to stigma or cost. Precision therapy, utilizing data from wearable devices and smartphones, enables the creation of personalized treatment plans tailored to everyone’s unique needs [62]. Moreover, AI-driven diagnostic systems are enhancing therapeutic interventions by providing mental health professionals with valuable insights derived from user data. Studies have shown impressive accuracy rates in predicting and classifying mental health conditions, such as depression and schizophrenia, with ML techniques achieving accuracies ranging from the low 60s to the high 90s. This improved diagnostic capability allows for earlier identification and intervention, which is crucial for effective treatment [63].

In addition to these advancements, smart devices and wearable technologies are playing an increasingly important role in monitoring mental health conditions. For example, the Q-sensor demonstrated an accuracy of 87% in detecting poor mental health and 78.3% for depression. These noninvasive, portable devices can seamlessly integrate into users’ daily lives, overcoming the resistance often faced by more traditional, invasive methods [64]. Furthermore, AI techniques are also advancing the detection and diagnosis of depression by leveraging various data sources, including audio, video, text, and physiological signals (4). This approach provides a more objective and accurate diagnosis compared to traditional subjective assessments. The integration of wearable devices enables the collection of large-scale psychophysiological data at a low cost, which is essential for training AI models (4). Ensemble methods have demonstrated significant improvements in detection accuracy over baseline methods, highlighting the potential of AI to reduce reliance on human subjectivity and enhance the overall effectiveness of depression detection [65].

### 2.3. Sensor Fusion and Multivariate Analytics

Combining data from diverse wearable sensors, also known as sensor fusion, allows even broader personalized health insights. Sensor fusion and multivariate analytics are crucial for enhancing the capabilities of AI-based sensors and biosensors in health analysis. By integrating data from multiple sensor sources, such as wearable devices and medical imaging, sensor fusion improves the accuracy and reliability of health monitoring systems, enabling more comprehensive insights than single-sensor data alone [66,67]. The application of AI algorithms significantly enhances the processing of this data, facilitating early disease prediction and timely clinical decision-making [11,46]. Moreover, frameworks like the AI-Based Body Sensor Network Framework (AIBSNF) propose systematic approaches to collect and analyze multivariate data, combining physiological signals with real-time location data for improved health outcomes [68]. However, challenges remain, including the need for organized data collection and the integration of diverse data modalities, which can complicate the analysis process [46]. The synergy between sensor fusion and AI analytics holds great promise for advancing personalized medicine and, as a result, improving healthcare delivery.

### 2.4. Case Examples of AI-Enabled Wearable Health Monitoring

Wearable sensors and biosensors integrated with ML have shown significant promise in various medical applications. For instance, the DOCTOR framework utilizes a multi-headed deep neural network to enable continual learning for multi-disease detection, allowing for the simultaneous classification of various diseases based on wearable medical sensor data and achieving superior accuracy compared to traditional methods [69]. Additionally, a study focused on detecting mental stress employed wearable physiological sensors (ECG, GSR, and skin temperature) and ML algorithms, demonstrating the potential for real-time stress monitoring and personalized interventions [70]. Furthermore, human activity recognition systems leverage wearable sensors and ML techniques, achieving classification accuracies of up to 95.78%, which is crucial for applications in elderly healthcare and smart homes [71]. Also, it should be noted that carbon nanotube-based biosensors trained with ML algorithms have been developed for the sensitive detection of malignant and nonmalignant cells, showcasing the versatility of ML in enhancing biosensor functionality [72]. In a recent study, Oliveira Filho et al. reported on the use of TinyML to remove background interference in complex solutions like cerebrospinal fluid. TinyML was implemented in low-power, portable systems for electrochemical applications, achieving high accuracy in discriminating between uric acid and ascorbic acid. The TinyML model reached an overall accuracy of 98.1% for a 32-bit float point unit and 96.01% after 8-bit quantization. These studies suggested that TinyML could enhance the reliability and real-time data processing abilities of future medical devices [57]. These examples illustrate the transformative impact of wearable sensors and ML in advancing healthcare monitoring and disease detection.

Wearable sensors and biosensors utilizing deep learning have shown significant promise in various medical applications. For instance, flexible wearable sensors have been developed to detect freezing of gait (FoG) in patients with Parkinson’s disease, employing a deep learning model that processes multi-modal sensory inputs to alert users and prevent falls [73]. In addition, a deep learning-enabled wearable device has been introduced for tracking movement disorders, achieving a high prediction accuracy for classifying different body postures, which is crucial for early diagnosis of neurological conditions [74]. Moreover, advancements in human activity recognition (HAR) systems have integrated deep learning techniques to monitor patient activities, aiding in the management of healthcare services and conditions such as stroke and epilepsy [75]. These applications highlight the transformative potential of wearable biosensors in enhancing patient care and monitoring, although challenges such as data accuracy and sensor integration remain to be addressed [76,77].

Wearable sensors and biosensors utilizing RNNs have shown significant promise in various medical applications, enhancing patient monitoring and diagnostics. These technologies leverage the ability of RNNs to process sequential data, making them ideal for real-time health assessments. RNNs have been effectively utilized in various healthcare applications. For example, wearable antennas and optimized recurrent neural networks (ORNNs) were used to enhance the medical communication process. A study focused on improving the quality of wireless communication in medical applications by investigating antenna S11 variation (AS11V) with harmonic suppression1. The researchers used a belt with a specific thickness and dielectric constants along with 3-short pin resonators to reduce unnecessary harmonics1. The ORNN approach demonstrated an accuracy of 99.17% in processing the collected data, making it highly effective for medical analysis [78].

Human healthcare from body sensor data and its practical applications in smart healthcare systems include wearable-based behavior recognition for patient rehabilitation. A study proposed a body sensor-based system for behavior recognition using deep RNNs, a promising deep learning algorithm for sequential information. Data from multiple body sensors, including an ECG, accelerometer, and magnetometer, was fused and enhanced using kernel principal component analysis (KPCA). The robust features were then used to train an activity RNN for behavior recognition. The system outperformed conventional approaches on three publicly available datasets, demonstrating its effectiveness [79].

In another work, a novel monitoring system was investigated using wearable sensors connected to a hospital database via IoT, with data classified by pre-convoluted fast recurrent neural networks (P-FRNN). The classification detected abnormal health data with improved accuracy and reduced time consumption and sent results to doctors when abnormalities were found. The simulation results were optimized, showing that P-FRNN achieved a comparable classification rate and low execution time [80]. Another interesting approach has been introduced by Mirto Musci et al., who developed the design of a software architecture based on RNNs for effective fall detection, running entirely on wearable embedded sensors. This study demonstrated that architectural minimization and accurate hyperparameter selection led to a workable model that compared favorably with other detection techniques [81].

Wearable sensors and biosensors utilizing Convolutional Neural Networks (CNNs) are revolutionizing healthcare by enabling real-time monitoring and analysis of health parameters. These technologies enhance patient care through various applications, demonstrating their effectiveness in diverse healthcare scenarios. For example, a CNN-based medical system that utilizes wearable sensors for the diagnosis of non-small cell lung cancer (NSCLC) was developed. The system achieved an accuracy rate of 0.84 with a dataset of 8000 case samples, providing valuable decision-making support for physicians based on patient data [82].

Moreover, a practical, wearable fall detection system that leverages Tiny Convolutional Neural Networks (TinyCNNs) on inertial sensors was investigated. The proposed TinyCNN achieved high accuracy and low latency in fall detection, making it suitable for real-life applications. The developed wearable system provides a practical solution for accurate and timely fall detection in everyday scenarios [83].

Wearable sensors and Long Short-Term Memory (LSTM) models are increasingly utilized in medical applications, enhancing patient monitoring and diagnosis. These technologies enable real-time health assessments and predictive analytics, significantly improving healthcare delivery. A study aimed to estimate the in vivo muscle forces occurring during human motion to understand motion control mechanisms and joint mechanics. It combined the advantages of CNNs and LSTM to propose a novel muscle force estimation method based on CNN–LSTM. A wearable sensor system collected kinematic data of hip, knee, and ankle joints during walking, which served as input for the neural network model. The CNN–LSTM model outperformed standard CNNs and LSTM in estimating muscle forces at various walking speeds, showing good robustness and generalization. This method provided a more convenient and efficient approach for clinical analysis and engineering applications compared to the SO method in OpenSim [84].

Wearable sensors and biosensors integrated with NLP are transforming medical applications by enhancing patient monitoring and data analysis. These technologies facilitate real-time health assessments and improve clinical decision-making through the interpretation of unstructured data. In some applications of NLP, we have a novel sensing system with NLP algorithms that is developed to improve communication in healthcare. The team designed CommSense to be used on mobile devices, like smartwatches, capturing patient/clinician interactions and processing them to extract key communication markers. They identified feasible communication metrics through a literature review and from consensus within the team. The software was developed using an existing Android smartwatch platform, incorporating sensors for physiological, gesture, and voice data. The pilot test involved simulated clinical scenarios to evaluate CommSense’s ability to extract communication metrics accurately [85].

## 3. Intelligent Point-of-Care Diagnostics

Smart PoC Diagnostics represents significant progress in healthcare delivery, combining rapid on-site testing with AI to enhance diagnostic accuracy and testing speed. This rapidly evolving field integrates state-of-the-art technologies for immediate sample analysis with advanced AI algorithms, enabling healthcare providers to make informed decisions quickly and with high efficiency. By leveraging ML and knowledge-based systems, these POCTs are changing patient care across various medical settings, from ERs to telemedicine, offering the potential for improved patient outcomes and more personalized treatment approaches. This section spotlights the POCT technologies and concepts and automated POCT, as well as some key cases that combine POCT with AI.

### 3.1. Point-of-Care Testing Technologies and Concepts

POCT technologies are transforming healthcare by enabling rapid diagnostics at or near the site of patient care. These innovations enhance accessibility, speed, and patient engagement [86,87,88]. PoC sensing technologies are revolutionizing diagnostics by enabling rapid on-site testing across various medical and environmental monitoring applications. These innovations leverage microfluidics, nanotechnology, and novel sensing materials to enhance sensitivity, speed, and user-friendliness [89,90,91].

**Figure 3:**
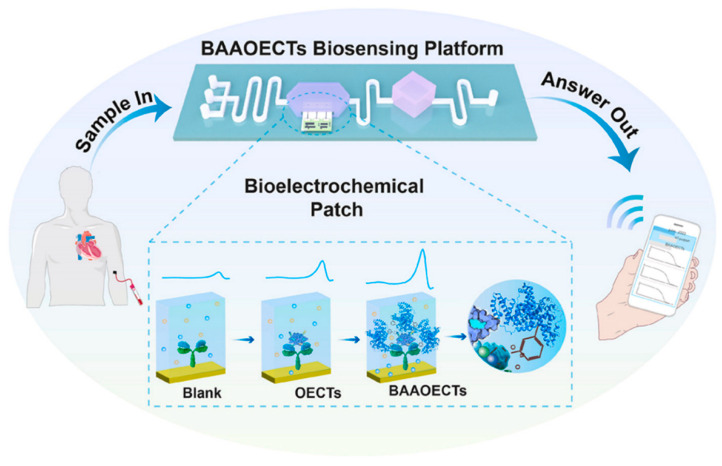
A compact and ultrasensitive bioelectrochemical patch was based on boronate-affinity amplified organic electrochemical transistors (BAAOECTs) for the POC sensing of glycoproteins. The figure is reused with permission from the publisher, Elsevier [92].

The development of biosensors for POCT applications significantly enhances health assessments at various locations, including bedside, home, and field settings. These biosensors offer rapid, accurate diagnostics, facilitating timely medical interventions. For bedside applications, devices like the boronate-affinity enhanced organic electrochemical transistor patch enable ultrasensitive detection of glycoprotein biomarkers, crucial for conditions such as heart failure, directly at the bedside (Figure 3) [92]. The developed bioelectrochemical sensing strategy exhibited a very low LOD of 300 aM in 25 min and was 1000× times more sensitive than the available commercialized kit tests. They proved that automatization with microfluidics, microcontrollers, and wireless sensing is possible and validated the PoC device for heart failure diagnosis. This PoC device showed great potential for broader glycoprotein detection applications and can be extended to address the need for sensitive, portable diagnostic tools in resource-limited settings.

The use of microfluidic chips allows for streamlined testing processes, ensuring quick results that can inform immediate clinical decisions. For home use, low-cost, portable biosensors facilitate at-home monitoring of chronic disease biomarkers, empowering patients to manage their health effectively without laboratory reliance. Innovations, such as smartphone integration for signal acquisition, enhance usability for non-professionals [93].

In field deployment, nanostructured biosensors enable rapid diagnostics in remote locations, addressing urgent health needs in underserved areas [94]. Field-effect transistor (FET)-based biosensors provide continuous monitoring, which is crucial for early disease detection in various environments [95]. While the advancements in PoC biosensors are promising, challenges remain in translating these technologies into widespread clinical use, particularly regarding regulatory approval and integration into existing healthcare systems [96]. These compact and portable devices facilitate immediate diagnostic capabilities, enhancing patient care by providing timely and precise information without centralized testing facilities.

### 3.2. AI for Automated Sample Analysis and Diagnostics

AI technologies are revolutionizing automated sample analysis and diagnostics across various medical fields. These systems enhance diagnostic accuracy and efficiency by integrating ML and explainable AI (xAI), particularly in complex areas like metabolomics and microbiology. The combination of AutoML and xAI has shown significant promise in cancer diagnostics, particularly in metabolomics. For instance, Auto-sklearn achieved an AUC of 0.97 for renal cell carcinoma (RCC) and 0.85 for ovarian cancer (OC), outperforming traditional ML methods. Shapley Additive Explanations (SHAP) were utilized to identify key metabolites, enhancing interpretability and clinical relevance [97,98].

Additionally, AI/ML-driven automated diagnostics streamline clinical resource management, allowing healthcare professionals to quickly identify diseases and improve patient outcomes. This technology reduces the time spent on routine tasks, enabling more focus on complex cases [99]. On the other hand, innovations in AI-controlled microfluidic devices have improved point-of-care testing reliability. These devices can autonomously manage fluid dynamics, significantly enhancing the accuracy of immunoassays [100].

### 3.3. Case Examples of AI-Empowered Point-of-Care Diagnostics

AI-empowered PoC diagnostics are revolutionizing healthcare by significantly improving the accuracy and efficiency of medical testing across various settings. These advancements utilize AI to aid healthcare providers in swiftly and effectively diagnosing conditions, especially in emergency and remote environments. Notable examples include AI-enabled ultrasound diagnostics, where deep learning models like MobileNetV2 and DarkNet53 achieve over 85% accuracy in interpreting ultrasound scans for conditions such as pneumothorax and hemothorax [101].

For instance, a novel AI-enabled device was developed for complete blood count (CBC) analysis, which can run multiple tests simultaneously, including a 3-part differential, using ML and deep learning for accurate cell classification, achieving high correlation coefficients with traditional laboratory methods [102]. Additionally, an AI-assisted framework for lung ultrasound scans aids less experienced clinicians in diagnosing pneumothorax by employing deep learning models for quality assurance and the lung sliding classification, achieving over 95% accuracy [103]. In another study, a PoC ultrasound (POCUS) has also been effectively utilized for diagnosing ventricular septal rupture, demonstrating AI’s capability to enhance diagnostic accuracy in emergency settings [104]. Furthermore, an AI-assisted mobile health system has been developed for the rapid detection of β-lactamase, a key factor in antimicrobial resistance, integrating a paper-based analytical device with a smartphone AI cloud for real-time error correction and result output [105].

Recently, Bhuyian et al. reported an AI-controlled microfluidic platform that was developed and operated via an Android smartphone based on an enzyme-linked immunosorbent assay (ELISA). Using region-of-interest (ROI) cascading and conditional activation algorithms, the platform incorporates a bubble trap to prevent false signals and control reagent movement. It successfully detected Human Cardiac Troponin I (cTnI) with a detection limit of 0.98 pg/mL, marking a significant step in the use of AI-based microfluidics for clinical diagnosis [100].

AI-enabled ultrasound diagnostics were involved in a recent study by Hernandez Torress and co-authors, where they used DL models, like MobileNetV2 and DarkNet53, to achieve over 85% accuracy in interpreting ultrasound scans for conditions such as pneumothorax and hemothorax [101]. Furthermore, the AI-assisted framework guides clinicians through lung ultrasound scans, achieving over 95% accuracy in detecting lung sliding, which is crucial for diagnosing pneumothorax [103]. In another work, an AI algorithm (AI-ECG) was applied to single-lead ECGs recorded during stethoscope exams as a potential PoC screening tool for left ventricular ejection fraction ≤40%. Conducted as an observational, prospective, multicenter study, AI-ECG was retrained to interpret single-lead ECGs from 1050 patients. The AI-ECG showed high performance, especially at the pulmonary valve position, with an AUROC of 0.85, sensitivity of 84.8%, and specificity of 69.5%. Combining outputs from two positions improved the area under the receiver operating characteristic curve to 0.91, sensitivity to 91.9%, and specificity to 80.2%. These results suggest AI-ECG’s potential for noninvasive, cost-effective PoC screening, enabling earlier diagnosis and treatment [106].

A supervised ML model was developed for pulmonary hypertension detection using noninvasive signals (orthogonal voltage gradient and photoplethysmographic) and a hand-crafted library of 3298 features. The model’s consistent performance across various demographics and its significant feature importance in conduction, repolarization, and respiration metrics highlight its potential for early detection and intervention in PoC diagnostic systems [107].

These key examples highlight the transformative potential of AI in enhancing PoC diagnostics and addressing challenges in accessibility and accuracy in healthcare delivery.

## 4. Opportunities and Challenges for AI in Personalized Medicine

### 4.1. Benefits of AI-Reinforced Wearable Sensors and Point-of-Care Testing

AI-reinforced wearable sensors and POCT offer transformative benefits in healthcare, significantly enhancing patient outcomes and healthcare efficiency. These advanced technologies enable continuous, real-time monitoring of vital signs and biochemical markers, providing critical data for early diagnosis and timely intervention. For instance, AI algorithms can analyze data from wearable sensors to detect anomalies and predict potential health issues before they become severe, promoting preventive healthcare [108]. This proactive approach reduces hospital admissions and healthcare costs by addressing conditions early. Moreover, AI integration enhances the accuracy and reliability of wearable sensors by filtering noise and extracting meaningful patterns from the data [108]. This is particularly beneficial in managing chronic diseases like diabetes, where continuous glucose monitoring through noninvasive wearable sensors can significantly improve patient comfort and compliance [109].

AI-driven analytics also facilitate personalized treatment plans by considering individual patient data, leading to more effective and tailored healthcare solutions [108]. In the context of POCT, AI-powered devices enable rapid and precise diagnostics at the patient’s location, reducing the need for laboratory visits and expediting treatment decisions [109,110]. This is crucial in emergency scenarios where a timely diagnosis can be life-saving. Additionally, the integration of AI with wearable sensors and POCT devices supports big data processing and real-time decision-making, enhancing the overall efficiency of healthcare delivery [108,111].

On the other hand, AI-aided POCT represents a significant advancement in healthcare technology with far-reaching social implications. By integrating AI with portable diagnostic tools, this innovation has the potential to democratize access to high-quality healthcare, particularly in remote and underserved areas. The ability to perform advanced diagnostics on-site allows for early detection of diseases, which is crucial for improving patient outcomes and reducing the overall burden on healthcare systems [14]. The personalization of treatment made possible by AI analysis of POCT data can lead to more effective medical interventions tailored to individual patient needs. This not only improves the efficacy of treatments but also potentially reduces adverse effects and the use of unnecessary medications. From an economic perspective, AI-aided POCT offers substantial cost savings by reducing the need for expensive laboratory tests and frequent hospital visits, making healthcare more affordable and accessible to a broader population [112]. Furthermore, this technology empowers healthcare providers by offering real-time insights and decision-making support. This is particularly valuable in resource-limited settings where specialist knowledge may not be readily available. By augmenting the capabilities of healthcare workers, AI-aided POCT can help bridge the gap in medical expertise between urban and rural areas by facilitating timely diagnostics without the need for extensive laboratory infrastructure [113,114].

The social impact extends beyond individual patient care. By improving overall public health through better disease management and prevention, AI-aided POCT can contribute to increased productivity and quality of life at a community level. It also has the potential to aid in the rapid response to disease outbreaks and pandemics by enabling quick, widespread testing and data collection [115].

AI is set to revolutionize POCT in the future by enhancing diagnostic accuracy and efficiency through advanced data analysis and pattern recognition, which can lead to more reliable results compared to traditional methods. The integration of AI with electronic health records will facilitate real-time data sharing, providing healthcare professionals with critical insights for better patient management. Additionally, AI-driven predictive models can help in forecasting disease progression, allowing for timely interventions and personalized treatment plans. As the POCT market continues to grow, the incorporation of intelligent technologies will not only improve the functionality of testing devices but also ensure that they remain economically viable and accessible to a broader patient population [116].

The synergy between AI, wearable sensors, and POCT represents a significant leap towards a more responsive, personalized, and efficient healthcare system, ultimately improving patient outcomes and quality of life.

### 4.2. Limitations of Artificial Intelligence in Point-of-Care Testing (POCT) Systems

The integration of AI into POCT systems, while promising for healthcare diagnostics, faces several significant challenges that limit its full potential. Technical barriers include the scarcity of high-quality training data, the opacity of AI decision-making processes, and difficulties in integrating AI with existing healthcare infrastructure. These technical challenges are compounded by regulatory hurdles, as agencies like the FDA continue developing frameworks for AI medical devices, and ethical concerns regarding patient privacy, data security, and algorithmic bias, particularly affecting underrepresented populations. This section discusses the main limitations that this field still faces in more detail.

#### 4.2.1. Validation and Regulatory Considerations for AI Diagnostics

As AI continues to revolutionize healthcare diagnostics, particularly in wearable sensors and POCT, ensuring the validity, safety, and regulatory compliance of these technologies becomes increasingly crucial. This section explores the key considerations and challenges in validating AI-powered diagnostic tools and navigating the complex regulatory landscape. Data quality and representation are essential for AI model development. Training and validation datasets must be diverse, accurately labeled, and potentially augmented to address class imbalances. Algorithm performance should be evaluated using appropriate metrics and compared to clinical gold standards. Clinical validation involves prospective studies, usability testing, and the assessment of impact on patient outcomes. Continuous monitoring and improvement are necessary to maintain performance and address potential biases [117]. Regulatory considerations for AI diagnostics include FDA pathways, EU MDR requirements, data privacy and security, and ethical implications. Adhering to these regulations ensures patient safety and trust in AI-powered tools. Challenges and future directions include regulatory harmonization, adaptive AI models, real-world evidence, and interdisciplinary collaboration. By addressing these challenges, stakeholders can help ensure that AI diagnostic tools are safe, effective, and trustworthy, ultimately improving patient outcomes and healthcare efficiency.

#### 4.2.2. Adoption and Implementation Challenges

The adoption and implementation of AI-reinforced wearable sensors and POCT face several significant challenges. Primarily, these challenges revolve around technology integration, cost, and data management, which can hinder the potential benefits of these innovations in healthcare. AI-enhanced wearable sensors and POCT systems necessitate seamless integration with existing healthcare infrastructures, including compatibility with various data formats and communication protocols, which can be complex and resource-intensive [22]. The development of multi-channel wearable sensors, such as those utilizing CRISPR/Cas12a for drug detection, showcases innovative solutions but also underscores the need for robust technological frameworks to support their deployment. POCT often incurs higher costs per test compared to traditional laboratory testing, limiting its adoption, especially in resource-limited settings [118]. While the integration of AI in wearable biosensors aims to reduce costs and improve efficiency, initial investments in technology and training remain significant barriers [46]. The vast amounts of data generated by AI and wearable sensors necessitate advanced data management systems. Issues related to data privacy and security are paramount, as sensitive health information is transmitted and stored [22].

There is a pressing need for quality management systems and guidelines to ensure the reliability and accuracy of POCT results [118]. Additionally, wearable sensors must consistently provide precise and reliable data to be useful in clinical settings, but factors like sensor placement, user movement, and environmental conditions can affect their performance [108]. Data privacy and security are critical concerns, as these devices collect sensitive health information that must be protected from unauthorized access. Integration with existing healthcare systems also poses a challenge requiring seamless data transfer and compatibility with various electronic health record systems [24,119].

The use of AI in POCT systems presents several limitations that can hinder its effectiveness. One significant challenge is the variability in user experience and training, which can lead to inconsistent results when using AI-driven devices in diverse testing environments [120]. Additionally, the evaluation protocols for AI systems often lack rigor, resulting in overestimated performance metrics that do not accurately reflect real-world applications [121]. Furthermore, the interpretability of AI algorithms remains a critical issue; clinicians may be hesitant to trust AI recommendations due to the “black-box” nature of these systems, which complicates their integration into clinical decision-making [122]. Lastly, the need for comprehensive evaluation methods that consider both diagnostic accuracy and the reasoning structure of AI systems is essential to ensure scalability and reliability in medical contexts. These limitations highlight the need for ongoing improvements in AI technology and its application in POCT.

The acceptance of AI-reinforced wearable sensors and intelligent POCT faces several challenges from both patients and medical doctors. Patients express concerns regarding the safety and reliability of AI technologies, fearing potential threats to their autonomy and increased healthcare costs, as well as issues related to data security and bias in data sources [123]. Additionally, the perception that AI may not adequately account for individual patient uniqueness contributes to resistance, particularly among those who view themselves as unique [124].

For medical professionals, the integration of AI into clinical practice requires significant buy-in, which is often hindered by a lack of understanding of AI’s role and effectiveness in enhancing patient care [125]. Furthermore, ethical considerations and the potential for security risks associated with connected devices pose additional barriers to acceptance [125,126,127]. Addressing these concerns is crucial for fostering trust and promoting the adoption of AI technologies in healthcare.

To address the challenge concerning gaining trust from both patients and doctors of these technologies, explainable AI (xAI) has emerged as a key solution, offering transparency and interpretability in AI decision-making processes. xAI plays a pivotal role in fostering trust and improving diagnostic processes in healthcare settings. By making AI systems more transparent, xAI bridges the gap between complex algorithms and human understanding, enhancing confidence in AI-driven insights [128]. This increased transparency is crucial for the acceptance and effective utilization of AI technologies in clinical settings, encouraging reliance on these insights while ensuring quality care [129]. Furthermore, xAI contributes significantly to improving diagnostic accuracy, particularly in applications such as disease detection and medical imaging, where wearable sensors and AI are integrated [130]. By addressing concerns about the “black-box” nature of traditional AI models, xAI enables better adoption and reliability in clinical practice. Moreover, the development of xAI techniques aims to enhance the interpretability of medical imaging and other healthcare applications, leading to improved diagnostic accuracy and patient outcomes [131].

One exemplary application of xAI in healthcare is the HealthxAI framework, which supports early diagnosis of cognitive decline in elderly individuals. This collaborative IoT system provides both numerical scores and natural language explanations for detected abnormal behaviors, making AI-driven assessments more accessible to caregivers and clinicians. By analyzing activities of daily living and locomotion patterns through smart home sensors, HealthxAI leverages well-known clinical indicators without requiring manual modeling or labeled datasets of abnormal behaviors. Extensive experiments with real-world data from 192 senior individuals demonstrated significant correlations between the system’s predictions and actual diagnoses, while a preliminary user study with clinicians showed improved task performance and increased trust in the system due to its xAI capabilities. xAI plays a pivotal role in making AI-driven healthcare technologies more understandable and reliable. By providing clear explanations of AI algorithms and their predictions, xAI contributes to the advancement of POCT and diagnosis, ultimately leading to better patient outcomes and more efficient healthcare delivery.

In general, to mitigate the challenges presented in this section, several methods can be employed. Implementing robust data security protocols, such as advanced encryption and anonymization techniques, can protect patient data and build trust in the system’s security. Regularly updating and validating AI algorithms can maintain their high accuracy and reliability, ensuring consistent performance. Providing comprehensive education and training for both patients and healthcare providers can increase understanding and trust in the technology, making users more comfortable with its use. Developing user-centered designs that are intuitive and user-friendly can enhance acceptance and ease of use, reducing barriers to its adoption [132].. Establishing channels for continuous feedback from users can improve the system based on real-world experiences, ensuring it meets patient needs effectively. By overcoming these challenges with targeted mitigation methods, the successful implementation of AI-reinforced wearable sensors and intelligent POCT can be significantly enhanced [117].

#### 4.2.3. Ethical Implications of AI in Personalized Medicine

A range of issues should be proactively addressed and managed to reduce and mitigate the ethical implications of AI in personalized medicine. One of the biggest concerns is the issue of privacy and data security, as AI systems rely on tremendous amounts of highly sensitive information about patients to deliver accurate and tailored medical treatments. There is always a potential for data breaches/leaks or misuse, which raises fears of stigmatization or discrimination [133,134]. In addition, there is always a risk of bias in AI algorithms, which is a critical ethical challenge as these models depend on the way they are trained for ML. This could lead to unequal treatment outcomes that could be based on gender, race, or socioeconomic status. Another ethical challenge in the use of AI technology in personalized medicine could include the informed consent of the patients in the use of these technologies, as many of them may struggle to understand how AI-driven decisions are made, thus affecting the ability of patients to make well-informed decisions. Furthermore, the question of accountability in the case of AI model errors or harm is of high importance, as there is always a chance of errors, thus making it difficult for liability.

Another interesting issue is access and equity, as there will be a risk that AI-driven personalized medicine could only allow preservation for those who can afford it, thus introducing more inequalities in healthcare. To strengthen trust in AI technologies and their implication in wearables and POCT, there should be rigorous validation, regulation, and ethical oversight to ensure that these novel systems are accurate, reliable, and aligned with the patient’s interests. Also, it should be noted that the role of physicians must remain integral to the decision-making process, ensuring that the technology complements, rather than replaces, the human effort in the healthcare system.

## 5. Conclusions

This review discussed the implementation of AI technology in POCT and wearable sensors, which has witnessed tremendous progress in recent years. This integration of AI with these tools leads to improvements in continuous health monitoring, real-time data processing, and rapid diagnostics. AI-empowered POCT devices provide rapid, accurate, and accessible diagnostics, which are very crucial for resource-limited settings and real-time decision-making. AI combined with wearable sensors allows for noninvasive monitoring of physiological health conditions and enhances personalized medicine choices. Despite the great advances in this field, the successful integration of these technologies still faces many challenges, including data privacy concerns and the challenges of getting regulatory approvals, as well as the need for robust AI algorithms that can accurately interpret highly complicated health data. Moreover, the integration of these new and advanced tools into the healthcare system requires careful consideration of user acceptance, cost-effectiveness, and data interoperability. Regardless of these challenges, the potential of AI-empowered wearable sensors and PoC diagnostics to take the healthcare system into the future is limitless, offering improved patient outcomes, enhanced effectiveness of data interpretation, and rapid decision-making. Future research is needed and should focus on addressing the above-mentioned challenges to ensure the reliability of AI-enabled health technologies, as well as exploring new pathways and opportunities for their full integration into personalized medicine.

## Figures and Tables

**Table 1:** The key review papers published recently in the field of biomedical research and AI.

Title	Main Points Discussed in the Published Review	Ref.
Revolutionary Point-of-Care Wearable Diagnostics for Early Disease Detection and Biomarker Discovery through Intelligent Technologies	Examines PoC systems and wearables for early disease detection and monitoring Discusses smart technology trends in clinical settings and biological assays Explores PoC systems and smart platforms for biomarker discovery Addresses technology translation from labs to broader applications Analyzes risks, biases, and challenges of AI integration in diagnostics Outlines prospects, challenges, and opportunities in the field	[20]
AI and the Internet of Medical Things (IoMT) Assisted Biomedical Systems for Intelligent Healthcare	Discusses AI’s role in enhancing IoMT and PoC devices for healthcare Covers AI applications in cardiac, cancer, and diabetes care Examines AI’s support in advanced robotic surgeries Analyze AI’s impact on IoMT device functionality and accuracy Addresses risk assessment in AI-powered medical devices Explores challenges and prospects of AI-integrated personalized IoMT Considers future directions for intelligent healthcare systems	[21]
Wearable AI biosensor networks	Reviews AI-assisted wearable biosensors for disease and fatigue monitoring Highlights the trend towards personalized, efficient, and accurate PoC diagnosis Notes need for further research on adaptive learning, synthetic data, and data privacy Discusses smartphone integration in biosensing systems Covers smartphone roles in sensor readout, data transfer, processing, storage, and display Emphasizes promising future due to increasing data capabilities and diverse functionalities	[22]
The Effectiveness of Wearable Devices Using AI for Blood Glucose Level Forecasting or Prediction: Systematic Review	Wearable devices with AI effectively forecast and predict BG in diabetics Studies reviewed were high quality but lacked diverse patient selection ML techniques, especially ensemble-boosted trees, show promise in BG forecasting Some studies reported high accuracy (e.g., 97% with support vector machines) Need for clearer distinction between “forecasting” and “prediction” in the literature Authors recommend further validation of commercial devices Wearable devices may potentially replace invasive glucose monitoring in future Review serves as key resource for advancing non-invasive diabetes management research	[23]
Recent Advances in AI and Wearable Sensors in Healthcare Delivery	AI and wearables transform healthcare into personalized, portable solutions Vital signs data analyzed with ML techniques Benefits: improved patient care, cost reduction, and enhanced clinical decisions Challenges: privacy, ethics, and AI model interpretation Identifies research gaps and future opportunities Emphasizes need for structured clinical data to avoid AI biases	[24]
Unlocking Tomorrow’s Health Care: Expanding the Clinical Scope of Wearables by Applying AI	Reviews AI-enabled wearables in cardiovascular medicine Covers smart watches, ECG patches, and smart textiles for various heart conditions Examines ML algorithm evolution in wearables Discusses validation frameworks and AI integration challenges Addresses fairness, equity, and user perspectives in development	[25]
A Systematic Review on the Advanced Techniques of wearable Point-of-Care Devices and Their Futuristic Applications	Review covers importance, design, and types of wearable sensors for POCT Highlights current breakthroughs in wearable integrated POCT devices Discusses present obstacles in the field Explores future potential, including IoT, for self-healthcare using wearable POCT	[26]
Where AI stands in the development of electrochemical sensors for healthcare applications: A review	Critical analysis of AI-assisted sensors and their specific tasks Data flow presentation: concept design to results for E-sensors Review of AI in wearable biomedical sensors Exploration of limitations in AI-assisted biomedical sensors Examination of the “promising” label in this context	[27]

## Data Availability

Data sharing does not apply to this article as no new data were created or analyzed in this study.
